# Microbiome engineering to enhance disease resistance in aquaculture: current strategies and future directions

**DOI:** 10.3389/fmicb.2025.1625265

**Published:** 2025-09-16

**Authors:** Muhammad Tayyab, Yongzhen Zhao, Yueling Zhang

**Affiliations:** ^1^Institute of Marine Sciences and Guangdong Provincial Key Laboratory of Marine Biology, Shantou University, Shantou, China; ^2^Guangxi Key Laboratory of Aquatic Genetic Breeding and Healthy Aquaculture, Guangxi Academy of Fishery Sciences, Nanning, China

**Keywords:** aquaculture microbiome, climate resilience, CRISPR engineering, disease resistance, fecal microbiota transplantation (FMT), multi-omics, probiotics, sustainable aquaculture

## Abstract

Aquaculture, a cornerstone of global food security, faces critical threats from disease outbreaks, antimicrobial resistance, and ecological disruption. Through a narrative analysis of over 160 studies, this review synthesizes advances in microbiome engineering—a sustainable approach to enhancing disease resistance in aquatic animals—addressing key gaps: the inconsistent efficacy of conventional probiotics and prebiotics under field conditions, and the need for climate-resilient solutions. Critically, we highlight the emergence of precision microbiome engineering as a transformative paradigm. We integrate findings from genomics, metabolomics, clustered regularly interspaced short palindromic repeats, and artificial intelligence to identify microbial strategies that enhance host resilience. Genomic and multi-omics methods reveal health-associated microbes and metabolites, such as *Vibrio*-dominated dysbiosis markers in shrimp and butyrate-mediated immunity. Guided by these biomarkers, we describe precision-tailored probiotics—host-derived or genome-edited *Bacillus subtilis* strains whose adhesion factors, metabolic outputs (e.g., butyrate, bacteriocins), and heat stress tolerance are matched to the target species’ gut niche. These are combined with complementary prebiotics (e.g., chitosan oligosaccharides) and synbiotics (e.g., *Lactiplantibacillus plantarum* plus king oyster mushroom extracts) that suppress pathogens through competitive exclusion and immune modulation. Ecologically rational innovations—interventions explicitly grounded in ecological theory (niche complementarity, K-selection) to stabilize resource-efficient microbiomes—such as fecal microbiota transplantation and synthetic consortia, demonstrate further disease control potential. Our synthesis reveals that translating microbiome engineering from laboratory to farm requires overcoming host-microbiome compatibility challenges and ecological risks. Policy alignment with the United Nations Sustainable Development Goals—Zero Hunger (Sustainable Development Goal 2), Climate Action (Sustainable Development Goal 13), and Life Below Water (Sustainable Development Goal 14)—is critical for sustainable adoption.

## 1 Introduction

Global aquaculture reached a historic milestone in 2022 by producing 94.4 million tonnes of aquatic animals, representing 51% of total production, and 130.9 million tonnes when including algae (FAO, 2024). This achievement highlights aquaculture’s crucial role in global food security, yet rapid expansion has intensified sustainability challenges such as disease outbreaks and antimicrobial resistance (AMR). Asia, responsible for 70% of aquatic animal production, faces severe disease-driven losses (Sampson, 2024). Despite vaccine development, pathogens like *Vibrio parahaemolyticus* (causing acute hepatopancreatic necrosis disease, AHPND) and *Streptococcus agalactiae* (causing streptococcosis) continue to cause global annual losses exceeding one billion dollars due to strain- and host-specific pathogenicity, inadequate infrastructure, and limited vaccine coverage in small-scale farms (Yen et al., 2021; Wang T. et al., 2024). These impacts are worsened in low- and middle-income countries (LMICs) by inadequate infrastructure and limited access to sustainable alternatives. The limitations of antibiotics and inconsistent probiotics spurred interest in holistic ‘microbial community management’ (Bossier et al., 2016), recognizing hosts as holobionts dependent on balanced microbiota. For example, a survey of 231 small-scale carp polyculture farms in Bangladesh revealed 46.8% report outbreaks of epizootic ulcerative syndrome (EUS) and columnaris, with an average mortality of 10.23% (Debnath et al., 2024). Similarly, a study of Lao PDR farms found 57.5% of fish farms rely on antibiotics against streptococcosis, with specialized operations exhibiting the highest antimicrobial dependence (Poupaud et al., 2022). Other threats include *Edwardsiella tarda* in Japanese eel and *Aeromonas hydrophila* in hybrid catfish, both causing 30–50% reductions in yield in intensive systems (Huang J. et al., 2024; Lin et al., 2024).

The reliance on antibiotics has fueled a global AMR crisis. In China, testing of 102 *V. parahaemolyticus* isolates from farmed shrimp found 46% resist multiple antibiotics, notably sulfamoxazole (56.9%) and erythromycin (33.3%) (Zhang F. et al., 2024). Meta-analyses reveal alarming trends: sulfonamide (sul1) and tetracycline (tetA) resistance genes dominate aquaculture systems, and groundwater antibiotic resistance gene (ARG) concentrations correlate with antibiotic use (Zainab et al., 2020). In Bangladesh, 97% of *E. coli* isolates from cultured fish were multidrug-resistant, carrying *blaTEM/blaCTX* genes (Rana et al., 2025), while 71.3% of foodborne *E. coli* strains in China showed tetracycline resistance (Liu C. et al., 2024). Chronic antibiotic exposure in aquatic environments amplifies environmental AMR risks by increasing resistance gene abundance (Chen Z. et al., 2025). Viral pathogens such as Decapod iridescent virus 1 (DIV1) exacerbate these problems by disrupting host microbiomes and risking spillover to wildlife (Wan et al., 2024).

Conventional alternatives like probiotics and prebiotics remain underutilized due to gaps in efficacy and knowledge. Probiotics often fail to consistently colonize the gut, while prebiotics lack pathogen-targeted precision (Guo et al., 2023). In Malaysia, 88.1% of shrimp farmers misunderstand AMR, and 50.5% use antibiotics prophylactically (Devadas et al., 2025), reflecting a critical need for effective alternatives. This necessitates a paradigm shift toward proactive ‘microbial education’ (Dantan et al., 2024) to establish resilient, health-promoting microbiomes early in development. The gut microbiome is a pivotal determinant of aquatic animal health. In grass carp, enrichment of SCFA-producing genera within *Lactobacillaceae* (e.g., *Lactobacillus*) and *Bacteroidaceae* (e.g., *Bacteroides*) correlated with upregulation of immune genes such as MHC2 and TNF-*α*; conversely, antibiotic-induced dysbiosis reduced microbial diversity and antioxidant capacity, triggering oxidative stress (Chen Z. et al., 2025).

Precision microbiome engineering addresses these limitations through targeted interventions that integrate ecological principles and functional enhancement. For example, CRISPR-edited *Cetobacterium somerae* XMX-1 knocks down viral receptors in zebrafish, reducing challenge-mortality by 75% (Liang et al., 2024). AI-designed synthetic communities (SynComs) that incorporate native *Photobacterium* spp. improve thermal-stress resilience (Toxqui-Rodríguez et al., 2025). Functional enhancement is evident in xylanase-expressing *Bacillus*, which elevates butyrate production in tilapia and activates immunity against *Aeromonas hydrophila* (Wang T. et al., 2024).

Despite these advances, key deployment barriers persist. These include (i) risks of horizontal gene transfer—such as temperature-amplified plasmid exchange in catfish systems (Li et al., 2025); (ii) host-specific microbiome variability that hampers generalizable formulations; (iii) regulatory inconsistencies across jurisdictions (Okoli et al., 2022; Rahayu et al., 2024); and (iv) socioeconomic constraints that limit access in low- and middle-income countries (LMICs) (Ghosh et al., 2022).

This review synthesizes advances in microbiome engineering—including next-generation probiotics, engineered synbiotics, fecal microbiota transplantation (FMT), and synthetic communities—to enhance disease resistance in aquaculture. Specifically, it explores CRISPR and AI-driven precision tools, identifies ecological, regulatory, and socioeconomic adoption barriers, and proposes a Sustainable Development Goals (SDG)-aligned roadmap targeting antimicrobial resistance reduction (SDG 3), food security (SDG 2), and marine biodiversity conservation (SDG 14). We emphasize that future advancements require tailored farm-specific probiotics, circular aquaculture systems, and global policy integration to ensure scalable, eco-safe aquaculture.

## 2 Literature search and study selection

We conducted a structured narrative review and searched Web of Science, Scopus, and PubMed for peer-reviewed studies published from January 2015 to August 2025 (last search: 11 August 2025). Search strings combined aquaculture terms with microbiome-engineering concepts using Boolean operators and truncation, for example: (aquaculture OR fish* OR shrimp OR prawn OR mollusc* OR mollusk*) AND (microbiome OR microbiota) AND (probiotic* OR prebiotic* OR synbiotic* OR postbiotic* OR FMT OR “fecal microbiota transplant” OR “faecal microbiota transplant” OR SynCom* OR “synthetic communit*” OR CRISPR OR gut-on-chip). Titles and abstracts, then full texts, were screened against inclusion criteria: (i) aquatic animals (finfish, crustaceans, mollusks); (ii) disease-resistance or immunity outcomes; and (iii) interventions involving probiotics, prebiotics/synbiotics, postbiotics, FMT, SynComs, or host/microbe engineering. We excluded non-primary studies (e.g., reviews, editorials, conference abstracts without data), terrestrial models, and interventions not targeting the microbiome from the primary synthesis. Relevant reviews and included studies’ reference lists were hand-searched to identify additional primary studies. Methods/tool papers (e.g., gut-on-chip) and non-aquatic models were cited for methodological context only and excluded from the primary synthesis. After deduplication, 162 primary intervention studies were included in the narrative synthesis.

## 3 Multi-omics insights guiding microbiome engineering

Recent multi-omics advances clarify host–microbiome–environment interactions by resolving microbial composition, function, and host responses at high resolution. These insights enable precision microbiome engineering, shifting from observation to targeted interventions in aquaculture. Integrating genomic, transcriptomic, metabolomic, and epigenetic findings with probiotic, prebiotic, and synbiotic applications illustrates how multi-omics accelerates microbiome engineering.

### 3.1 Genomic and metagenomic approaches

Genomic and metagenomic approaches decode host-microbiome-environment interactions by mapping microbial community dynamics under health, disease, or stress. These methods identify keystone taxa (e.g., opportunistic pathogens like *Aeromonas*) and functional shifts linked to dysbiosis, enabling targeted interventions. High-throughput sequencing (e.g., 16S rRNA gene profiling, shotgun metagenomics) enhances resolution of microbial profiles across host health and environmental gradients. These approaches identify key microbial players such as opportunistic pathogens (e.g., *Aeromonas*, *Vibrio*) and beneficial taxa (e.g., *Cetobacterium*, *Weissella*), with abundance shifts strongly correlating to host status. Critically, functionality is strain-specific: pathogenic potential varies within *Aeromonas*/*Vibrio*, and probiotic properties are not universal in *Cetobacterium*/*Weissella* (Wang T. et al., 2024; Li et al., 2025). Metagenomics facilitates pathogen discovery and dysbiosis-disease linkages, exemplified by metabarcoding identifying a novel *Flavobacterium* species causing peracute skin disease in rainbow trout, distinct from classical columnaris strains (Zamparo et al., 2025). Viral profiling through virome analyses detects pathogens like white spot syndrome virus in environmental reservoirs and novel caliciviruses linked to mass fish mortality (Mercer et al., 2024; Su et al., 2024). Advanced techniques such as Oxford Nanopore sequencing enhance resolution in complex matrices like fish mucus (Domingo-Bretón et al., 2024). However, these DNA-centred tools have well-recognized constraints: short-read assemblies can mask low-abundance taxa and hamper strain-level resolution; draft metagenomes rely on gene annotations that are predictive rather than experimental; and high host DNA backgrounds can dilute microbial signals, particularly in gut, gill, or skin biopsies. Stable isotope probing (DNA-SIP/RNA-SIP) and long-read sequencing are now being combined with metagenomics to assign functional genes to active taxa and partially alleviate these blind spots (Alcolombri et al., 2022; Han Y. et al., 2024). Spatial heterogeneity is critical, with distinct microbiomes inhabiting mucosal surfaces (gill, skin, gut, ovary). For instance, the gut of olive flounder harbors more antibiotic resistance genes and *Vibrionaceae* than functionally diverse gill/skin communities, underscoring the need for site-specific probiotics (Yu et al., 2025).

### 3.2 Metabolomic insights into host-microbiome crosstalk

Metabolomics reveals how microbial metabolites (e.g., SCFAs) mediate host-microbe crosstalk, influencing immune pathways and stress resilience. This mechanistic insight identifies therapeutic targets for precision engineering. For example, butyrate and other SCFAs serve dual roles as enterocyte energy sources and immunomodulators, acting via histone deacetylase (HDAC) inhibition or G-protein-coupled receptor signaling. Dietary interventions, such as supplementation with *Clostridium butyricum* in shrimp, elevate beneficial metabolites and enhance mucosal immunity and pathogen resistance (Li S. et al., 2022; Liao et al., 2023). Yet metabolite profiles alone seldom reveal which organism produced a given compound. Emerging compound-specific stable isotope labeling (e.g., ^13^C or ^15^N SIP) tracked by high-resolution MS, as well as spatial metabolomics coupled with fluorescence *in situ* hybridization (FISH-SIMS), now help connect metabolite fluxes to specific microbial producers (Uengwetwanit et al., 2020; Alcolombri et al., 2022). Beyond SCFAs, metabolomics detects broader shifts in bile acid and amino acid metabolism that contribute to immune resilience. Critically, metabolomics reveals pollutant-induced dysbiosis, where microplastics, pesticides (e.g., deltamethrin), and polychlorinated biphenyls disrupt gut-liver axes. These pollutants alter lipid metabolites like lysophosphatidylcholines, trigger oxidative stress, and dysregulate signaling pathways (e.g., PPAR, apoptosis genes), detectable through integrated metabolomic analyses (Song et al., 2025; Zhong et al., 2025).

### 3.3 Transcriptomic and epigenetic regulation

Transcriptomics and epigenetics uncover host response mechanisms to microbiome shifts, including immune gene regulation and epigenetic priming of ‘trained immunity’—even in invertebrates lacking adaptive immunity. Nevertheless, transcript counts rarely translate directly into protein function; low-expression genes may be missed, and host RNA often dominates libraries. Coupling metatranscriptomics with metaproteomics or ribosome profiling can overcome these bottlenecks by verifying actual protein synthesis (Zhao C. et al., 2023; Heyer et al., 2025). Probiotics such as *Lactiplantibacillus plantarum* and microbial metabolites like butyrate upregulate immune genes (e.g., *proPO*, *lysozyme*, *IL-10*, antimicrobial peptides) and pathways (Toll, Imd, NLRP3 inflammasome), enhancing defense mechanisms (Shan et al., 2021; Wang T. et al., 2024; Guzman et al., 2025). Under environmental stress, transcriptomics uncovers conserved response pathways: in Pacific white shrimp exposed to heat stress, it reveals energy repartitioning through glycolysis, immune modulation via C-type lectin and IL-17, and glutathione-mediated antioxidant defense (Liu et al., 2025). Similarly, ammonia stress in fish disrupts amino acid metabolism and activates apoptosis pathways (Peng et al., 2025). Epigenetic mechanisms mediate microbiome effects on immunity; butyrate, as an HDAC inhibitor, alters chromatin accessibility (e.g., enhancing *IL-17D* expression in tilapia for neutrophil recruitment) and influences DNA methylation. Microbiome-induced “trained immunity” occurs even in invertebrates lacking adaptive immunity through epigenetic priming (Liao et al., 2023; Wang T. et al., 2024; Guzman et al., 2025). Gnotobiotic models confirm that microbiome composition directly shapes host physiology, including immune modulation and spatial microbial distribution (Adade et al., 2023).

### 3.4 Integrated multi-omics for precision engineering

Integrated multi-omics bridges microbial composition, host physiology, and environmental interactions to identify biomarkers (e.g., microbial ratios signaling dysfunction) and refine precision strategies. This integration yields systems-level insights linking microbial dynamics, host physiology, and environmental interactions. It identifies robust biomarkers, such as genus-level microbial ratios (e.g., *Vibrio*/*Photobacterium* in shrimp) signaling stress-associated dysbiosis and immune disruption, and unravels complex mechanisms. For example, multi-omics links *Vibrio* proliferation to glutathione depletion and immune dysregulation specifically in shrimp under combined ammonia/salinity stress, and *Vibrionaceae* increase (with a shift in commensal genera) in seabream during parasitic infection (Li et al., 2025; Toxqui-Rodríguez et al., 2025). Because each single-omics layer is imperfect, integrated designs (e.g., genome-resolved metaproteomics, metabolite SIP linked to metagenome-assembled genomes [MAGs]) are indispensable for triangulating taxon→gene expression→metabolite relationships and thereby closing attribution gaps highlighted above (Rasmussen et al., 2022; Hansen et al., 2023). These insights directly inform precision strategies: optimizing probiotic formulations (e.g., *Lactococcus lactis* D1813 for specific salinity/dissolved oxygen conditions), designing targeted dietary supplements (e.g., antioxidants based on stress pathways), and developing novel therapies like antimicrobial peptides (e.g., Lvvibriocin-GK). Omics reveals that such peptides reduce pathogen loads, stimulate immunity (e.g., lysozyme activity), modulate microbiota, and suppress inflammation (Adil et al., 2025; Sun et al., 2025). Nutraceuticals like astaxanthin further mitigate tissue damage by regulating apoptosis and metabolism genes (He et al., 2025). Collectively, multi-omics underpins three synergistic pillars of microbiome engineering: identifying biomarkers to inform probiotic/synbiotic design; leveraging omics-derived targets (e.g., butyrate-producing taxa) for pathogen suppression; and using transplantation strategies (e.g., synthetic consortia) for ecological restoration. This closed-loop system—where omics guides intervention design and outcomes refine models—enables sustainable disease management in aquaculture.

### 3.5 Probiotics, prebiotics, and synbiotics as engineering tools

**Figure 1:**
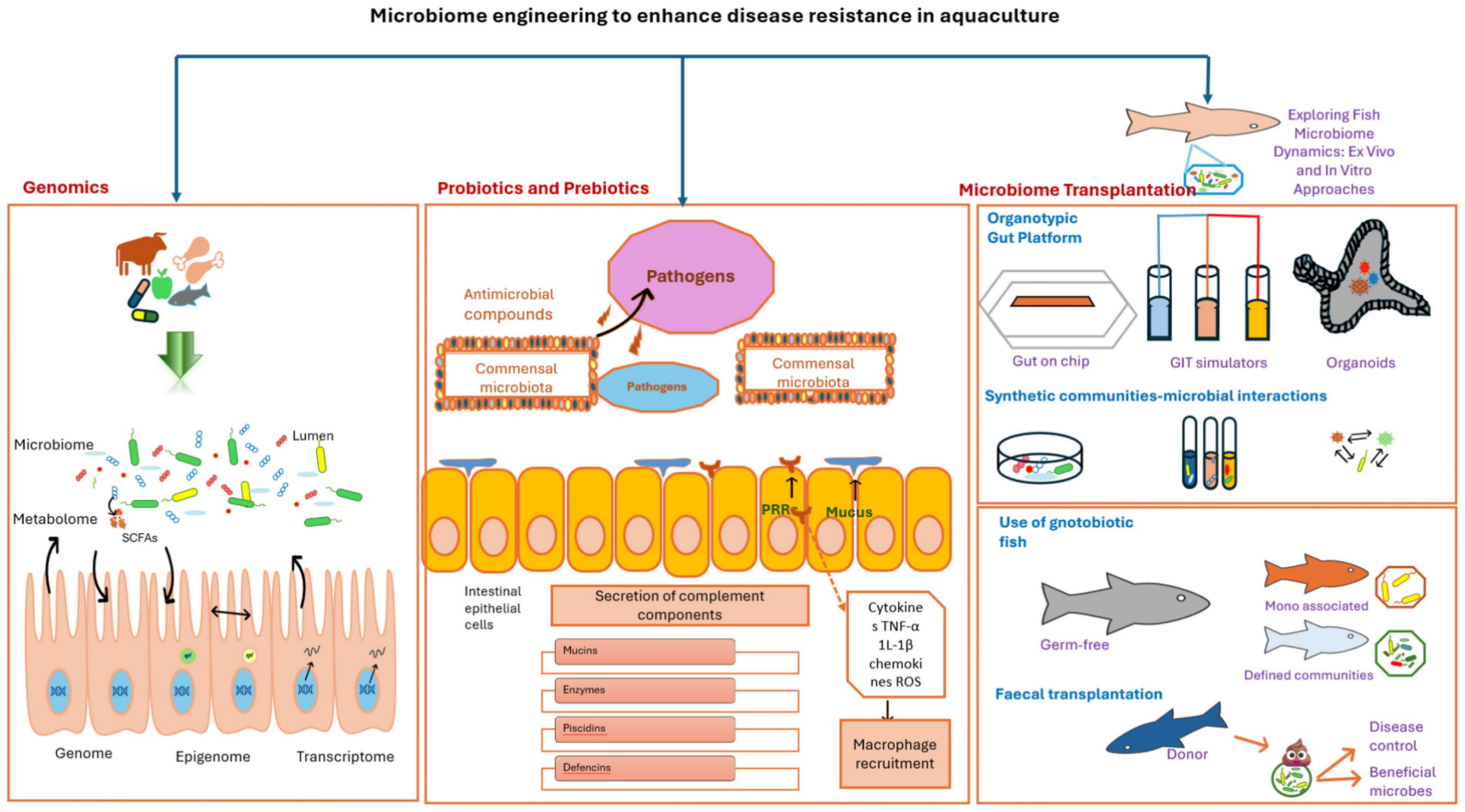
Integrated conceptual framework of microbiome engineering strategies for enhancing disease resistance in aquaculture. The framework unifies three synergistic pillars: Multi-omics approaches (left) dissect microbial community dynamics, host–microbe interactions, and functional pathways (e.g., immune modulation, stress response) through genomics, transcriptomics, metabolomics, and epigenomics. These insights identify therapeutic targets (e.g., butyrate-producing bacteria) and dysbiosis biomarkers. Probiotics and prebiotics (center) deploy antimicrobial compounds (e.g., bacteriocins), engineered microbial consortia (e.g., *Bacillus* spp.), and host-targeted strategies (e.g., barrier reinforcement) to exclude pathogens (e.g., *Vibrio*, *Aeromonas*) and enhance resilience. Microbiome transplantation (right) restores dysbiotic microbiomes via fecal microbiota transplantation (FMT), rationally designed synthetic communities (SynComs), or in vitro platforms (e.g., gut-on-chip), validated through gnotobiotic models (germ-free hosts colonized with defined microbial communities). Bidirectional arrows illustrate functional synergy: multi-omics guides probiotic and SynCom design; transplantation efficacy is monitored via omics; and probiotics/transplantation generate data to refine omics models. Together, these pillars enable pathogen suppression, immune enhancement, and ecological stability while reducing antibiotic dependence.

A key premise in probiotic development is that strain-specific traits (e.g., spore formation, bacteriocin production) must align with host physiology and environmental conditions for efficacy. Probiotics exert their protective effects in aquaculture through multifaceted mechanisms, including the production of antimicrobial compounds (e.g., bacteriocins, organic acids, enzymes), competitive exclusion of pathogens, and immunomodulation (Figure 1; Table 1). These mechanisms are harnessed in microbiome engineering to design targeted interventions, though their efficacy depends critically on strain selection, host compatibility, and environmental stability. For instance, *Bacillus subtilis* subsp. *inaquosorum* BSXE-2102 synthesizes 12 secondary metabolites with potent antagonistic activity against aquatic pathogens (Qin et al., 2025), while *Lactobacillus plantarum* strains from kefir produce bacteriocins that suppress *Vibrio alginolyticus* (Tseng et al., 2023). Strain-specific efficacy is evident in *Bacillus velezensis* FiA2, which produces broad-spectrum oxydifficidin (Khan et al., 2025), and *Streptomyces* sp. D-6, which delivers novel bioactive metabolites (Zhao et al., 2025). Meta-analyses confirm *Bacillus* spp. outperform *Lactobacillus* in pathogen inhibition due to spore-forming resilience, whereas *Streptomyces* strains show unique bioremediation potential (Giri et al., 2024; Hoseinifar et al., 2024). However, limitations persist: survivability under gastrointestinal stress varies (31–75% acid tolerance at pH 2–4), adhesion capacity to the GI tract (a critical determinant of colonization success) is often strain-specific, efficacy is host-dependent, and inconsistent trial results occur across environments or host genotypes. For example, *Bacillus subtilis* NTU-18 improves growth in *Anguilla japonica* glass eels but requires precise dosing for other species (Lin et al., 2024). These constraints highlight the need for engineered solutions, such as encapsulation or host-adapted consortia, to improve reliability.

**Table 1:** Pathogen-specific microbiome interventions in aquaculture.

Pathogen	Host species	Intervention type	Key outcome	Efficacy trend	Clinical implication	Reference
*Vibrio harveyi*	Grouper (*Epinephelus* spp.)	Engineered probiotic (*Bacillus subtilis* + FIgE antigen)	63% survival; ↓ *Vibrio* abundance	High efficacy (antigen-specific)	Precision engineering enhances pathogen targeting	Hou et al. (2025)
*Vibrio parahaemolyticus*	Rabbitfish (*Siganus canaliculatus*)	Synbiotic (*Clostridium* + polysaccharide)	Enhanced resistance; ↑ antioxidant capacity	Moderate-high (holistic support)	Prebiotics boost probiotic functionality	Bakky et al. (2025)
*Aeromonas hydrophila*	Yellow catfish (*Pelteobagrus fulvidraco*)	Microbial shift analysis	Dysbiosis (↑ *Akkermansia*, ↓ *Plesiomonas*) linked to immune suppression	Diagnostic insight	Early dysbiosis as biomarker for infection risk	Ci et al. (2024)
*Vibrio* spp.	Pacific white shrimp (*Litopenaeus vannamei*)	Synbiotic (*Lactobacillus* + plant extract)	72% survival; ↑ digestive enzymes	High efficacy	Plant extracts enhance probiotic colonization	Phan et al. (2025)
*Vibrio harveyi*	Pearl gentian grouper (*Epinephelus* spp.)	Pathogen-induced dysbiosis study	Immune dysregulation (↑ *Sphingomonas*, ↓ *Lactobacillus*)	Mechanistic insight	Dysbiosis precedes clinical disease	Wang F. et al. (2024)
*Aeromonas hydrophila*	Rohu (*Labeo rohita*)	Synbiotic (*Bacillus* + fructooligosaccharide)	↑ Survival; ↑ hematological parameters	Consistently effective	Synbiotics outperform monostrain probiotics	Sukul et al. (2023)
*Vibrio parahaemolyticus*	White shrimp (*Penaeus vannamei*)	Synbiotic (*Leuconostoc* + dextran)	68% survival; ↑ immune genes	High efficacy	Prebiotics enhance immunomodulation	Huang et al. (2023)
*Yersinia ruckeri*	Rainbow trout (*Oncorhynchus mykiss*)	Synbiotic (*Pediococcus* + flavonoids)	Minor ↑ disease resistance; improved lipid use	Low-moderate	Better for metabolic health than pathogen control	Villumsen et al. (2020)
*Aeromonas hydrophila*	Nile tilapia (*Oreochromis niloticus*)	Synbiotic (*Pediococcus* + pistachio polysaccharide)	40% ↓ mortality; ↑ antioxidants/gut barrier	High efficacy	Polysaccharides critical for gut integrity	Mohammadi et al. (2022)
*Vibrio alginolyticus*	White shrimp (*Litopenaeus vannamei*)	Synbiotic (*Lactobacillus* + mushroom extract)	↑ Survival; ↑ phenoloxidase/lysozyme	High efficacy	Fungal extracts amplify immune activation	Prabawati et al. (2022)
White Spot Syndrome Virus	White leg shrimp (*Litopenaeus vannamei*)	Synbiotic (*Pseudoalteromonas* + fructooligosaccharide)	52.8% survival post-coinfection	Moderate efficacy	Limited protection against viruses	Nababan et al. (2022)
Mercury toxicity	Common carp (*Cyprinus carpio*)	Selenium-enriched *Bacillus subtilis*	↓ *Aeromonas*; ↓ inflammation	Adjunctive therapy	Probiotics mitigate non-infectious stressors	Shang et al. (2022)
*Streptococcus agalactiae*	Nile tilapia (*Oreochromis niloticus*)	Synbiotic (*Lactobacillus* + watermelon rind)	68% survival; ↑ mucosal immunity	High efficacy	Agricultural waste valorization enhances accessibility	Van Doan et al. (2021)

↑ indicates an increase; ↓ denotes a decrease.

#### 3.5.1 Probiotic mechanisms and strain selection

Host genetic background critically modulates probiotic interactions. This host-specificity underscores the need for precision engineering: Genetically selected gilthead sea bream (*Sparus aurata*) exhibit enhanced intestinal barrier function with *Bacillus*-based probiotics, while non-selected strains show reduced performance (Naya-Català et al., 2024). Similarly, hybrid grouper displays genotype-dependent immune responses to *Exiguobacterium acetylicum* G1-33, with optimal dosing (10^8^ CFU/g) improving survival against *Vibrio harveyi* by 72% (Zhang M. et al., 2024). Variable colonization across species (e.g., *Pediococcus acidilactici* efficacy in salmonids but not non-salmonids) further necessitates host-adapted formulations (Soto-Dávila et al., 2024).

Probiotics competitively exclude pathogens by colonizing mucosal surfaces and consuming essential nutrients (Table 2). Ecological strategies based on promoting K-selected microbial communities—characterized by stability and resource efficiency (typical of slow-growing, competitive species)—over opportunistic r-strategists (fast-growing species adapted to unstable environments) enhance colonization success and pathogen exclusion (Vadstein et al., 2018). This principle is demonstrated in a study where dietary *Bacillus subtilis* BSXE-1601 enhanced disease resistance of *Penaeus vannamei* against *Vibrio parahaemolyticus* and significantly modulated the rearing water microbiota, reducing its overall diversity and ecological network complexity while increasing the relative abundance of the genus *Marivita*, a member of the often beneficial *Rhodobacteraceae* family (Luo et al., 2024a). Effective engineering prioritizes strains with niche competence (e.g., *Bacillus aryabhattai* CKNJh11 biofilm formation) and complementary functions (Dangsawat et al., 2025). Concurrently, probiotics prime immune responses: SYNLAC Prime upregulates serine protease (*SP*), prophenoloxidase (*proPO*), and peneidin genes in shrimp (Cheng et al., 2024), while *Leuconostoc mesenteroides* B4 with dextran activates Toll and Imd pathways (Huang M. Y. et al., 2024). Immunomodulatory secretion—decoupled from live-cell requirements via postbiotics (e.g., *B. subtilis* AAHM-BS2360 inducing lysozyme activity)—offers alternative engineering avenues (Wiratama et al., 2025).

**Table 2:** Mechanisms and comparative efficacy of probiotics in aquaculture.

Intervention type	Probiotic strain	Host species	Primary mechanism (s)	Target pathogen	Outcome	Efficacy rating	Key insight	Reference
Postbiotic	*Bacillus subtilis* (postbiotics)	*Labeo rohita* (rohu)	Antibacterial activity, immune modulation	*Vibrio* spp.	Reduced pathogen colonization; enhanced immune response.	High	Postbiotics bypass colonization challenges; ideal for antibiotic-restricted systems.	Kumar et al. (2025)
Probiotic-only	*Exiguobacterium acetylicum* G1-33	Hybrid grouper	Gut morphology enhancement, immune gene upregulation	*Vibrio harveyi*	72% survival improvement (optimal at 10^8^ CFU/g).	High	Dose-dependent efficacy; critical for precision dosing.	Zhang M. et al. (2024)
*-*	*Bacillus velezensis*	Pacific white shrimp	Digestive enzyme stimulation, antioxidant activity	General pathogens	Enhanced growth, survival, and immunity; reduced oxidative stress.	High	Multi-functional: Combines growth promotion + pathogen defense.	Abdelsamad et al. (2024)
*-*	*Bacillus subtilis* strains (6–3-1, HAINUP40)	Hybrid grouper	Lipid metabolism modulation, antioxidative activity	*Vibrio harveyi*	Strain-specific growth promotion; stress resilience.	Moderate-High	Strain specificity impacts outcomes; requires host-matched formulations.	Han C. et al. (2024)
*-*	*Streptomyces* spp.	Fish and shellfish	Antibiotic production, quorum sensing inhibition	*Aeromonas*, *Vibrio*	Improved disease resistance via antimicrobial metabolites.	Moderate	Bioremediation potential; reduces need for chemical treatments.	Butt et al. (2024)
*-*	*Bacillus subtilis* YBS29	Indian major carp	Bacteriocin production, competitive exclusion	*Aeromonas veronii*	Reduced mortality; safe for aquatic use.	High	Safe alternative to antibiotics; no adverse effects reported.	Paul and Rahman (2022)
*-*	*Lactiplantibacillus plantarum*	Freshwater fish	Antimicrobial activity, gut adhesion	*A. salmonicida*, *E. coli*	Enhanced disease resistance in cold/warm water systems.	High	Broad thermal adaptability; suitable for diverse climates.	Iorizzo et al. (2022)
*-*	*Bacillus velezensis* WLYS23	Snakehead fish	Antimicrobial peptide synthesis	*Aeromonas* spp.	Improved survival; safe application.	High	Novel peptide mechanism; high pathogen specificity.	Zhang et al. (2021)
*-*	*Pseudomonas putida*	Nile tilapia	Immune response stimulation	*Aeromonas hydrophila*	Increased survival post-challenge.	Moderate	Immunomodulation-focused; less effective for severe outbreaks.	Abomughaid (2020)
Synbiotic	*Bacillus subtilis* + seaweed extract	Nile tilapia	Immune modulation, anti-inflammatory regulation	*Aeromonas hydrophila*	Improved water quality, growth, and resistance.	High	Synergistic combo outperforms monostrain probiotics.	Radwan et al. (2024)

#### 3.5.2 Host-adapted and precision formulations

Prebiotics, such as chitosan oligosaccharide (COS), selectively stimulate beneficial gut bacteria. COS supplementation in *Penaeus vannamei* enriches beneficial bacteria such as *Algorimicrobium* and *Roseibium* while suppressing pathogenic *Vibrio* spp., including *V. parahaemolyticus* and *V. rotiferianus* (Fu C. et al., 2025), and fermented pomegranate peel polyphenols elevate beneficial genera including *Lactobacillus* and *Bifidobacterium* (Yu et al., 2024). These shifts enhance gut barrier function but are dose-sensitive; high resistant starch (3%) disrupts microbiota balance in *Micropterus salmoides* (Zhang X. et al., 2025). Prebiotics also enhance innate immunity: dietary piperine elevates superoxide dismutase (SOD) and glutathione peroxidase (GPx) in shrimp (Albaqami, 2024), while tea polyphenols mitigate enteritis in grass carp by suppressing NF-κB and activating Nrf2/Keap1 pathways (Ma et al., 2025). However, prebiotics alone often fail to sustain microbial shifts without probiotics, highlighting the need for integrated approaches.

#### 3.5.3 Prebiotics and Postbiotics for targeted modulation

Postbiotics—*“preparations of inanimate microorganisms and/or their components that confer a health benefit on the host”* (Salminen et al., 2021)*—*are emerging as a practical alternative to live probiotics in aquaculture. Because the cells are non-viable, postbiotics tolerate pelleting temperatures, avoid horizontal gene-transfer risks, and typically face a lighter regulatory burden (Vinderola et al., 2022; Gervasoni et al., 2023). Their bioactivity arises from (i) immunomodulatory cell-wall fragments that up-regulate TLR-dependent NF-κB/MAPK signalling and boost plasma IgM (↑ 37% in Atlantic salmon; Table 2) (Kumar et al., 2025); (ii) secreted antimicrobial peptides that competitively exclude pathogens such as *Vibrio* spp. (↓ 60% colonisation in shrimp) (Sudhakaran et al., 2022); and (iii) metabolites that strengthen epithelial barriers by inducing mucin genes and antioxidant enzymes (Kavita et al., 2024). Comparative trials show postbiotics can out-perform their live counterparts, delivering 40–65% higher survival against WSSV and lowering production costs by ~30% (Abdel-Latif et al., 2022; Thorakkattu et al., 2022).

#### 3.5.4 Synbiotic strategies and system-level optimization

Synbiotics—rational combinations of probiotics and prebiotics—are formally categorized as either *complementary* (independent mechanisms) or *synergistic* (prebiotics selectively enhancing co-administered probiotics) under the updated ISAPP framework (Swanson et al., 2020). In aquaculture, synergistic formulations are prioritized to overcome individual limitations of probiotics (e.g., survivability) and prebiotics (e.g., transient effects), representing a cornerstone of microbiome engineering. For example, *Lactobacillus plantarum* 7–40 with king oyster mushroom extract (KOME) enriches lactic acid bacteria, reduces *Vibrio* counts, and upregulates immune genes (*proPO*, *lysozyme*, *crustin*), achieving 72% survival against *V. alginolyticus* in shrimp (Prabawati et al., 2022). The synergy arises from prebiotics enhancing probiotic survival/metabolic activity (e.g., KOME stimulating *L. plantarum* growth) and probiotics metabolizing prebiotics into immunomodulatory compounds like short-chain fatty acids (Kewcharoen and Srisapoome, 2022). Commercial-scale trials confirm synbiotic efficacy: *Bacillus subtilis* and *Lactococcus lactis* PH3-05 improved survival of tropical gar larvae by 46% (Prabawati et al., 2022), while *Bacillus* spp. + BiOWISH Feedbuilder Syn3 (BiOWiSH Technologies, Cincinnati, OH, USA) boosted Nile tilapia survival by 20% in biofloc systems (Oliveira et al., 2025). Encapsulation technologies (e.g., alginate-microencapsulated *B. licheniformis* with >90% gastric viability) further stabilize synbiotics for targeted delivery (Cota-Gastélum et al., 2025). These studies underscore the potential of synbiotics to optimize microbiome engineering in aquaculture. Meta-analyses confirm that biofloc systems—0inherently synbiotic environments – consistently enhance key immune parameters (e.g., lysozyme, immunoglobulins, antioxidant enzymes) and disease resistance across aquatic species (Khanjani et al., 2023). The host-specificity and environmental constraints of conventional probiotics underscore the need for precision solutions. FMT and SynComs address these gaps by restoring or designing communities tailored to host ecology. When resident microbial communities are deeply disrupted, whole-community approaches—fecal microbiota transplantation and purpose-built synthetic consortia—offer a broader remedy.

## 4 Microbiome transplantation and synthetic communities

As scalable implementations of precision engineering, FMT and SynComs leverage multi-omics insights to reintroduce keystone taxa or synthetically assemble consortia that optimize host functions beyond restoration. Transplantation strategies are posited to restore dysbiotic ecosystems by reintroducing keystone taxa, with their success largely contingent on donor-recipient compatibility. Standardized protocols for FMT—such as 7-day antibiotic decontamination followed by 3 weekly transplant doses—significantly improve microbial engraftment and stability in aquatic models, as demonstrated in murine studies adapted for aquaculture applications (Amorim et al., 2022). However, optimal antibiotic pre-treatment durations (ranging from days to weeks) and dosing frequencies (single to weekly administrations) remain species-dependent and require further refinement (Marclay et al., 2022; Han Z. et al., 2024; Karimianghadim et al., 2025). Long-term engraftment viability depends on species-specific factors, microbiota composition, and environmental conditions (Han Z. et al., 2024).

### 4.1 Fecal microbiota transplantation

Microbiome transplantation strategies represent two distinct approaches for enhancing aquaculture sustainability. Fecal microbiota transplantation (FMT) leverages naturally evolved communities from healthy donors to restore dysbiotic hosts, functioning primarily as a restorative intervention (El-Son et al., 2025). In contrast, synthetic microbial communities (SynComs) employ rationally designed, precision-engineered consortia of defined beneficial strains to modulate host phenotypes beyond restoration (Guo et al., 2023; El-Son et al., 2025).

FMT has demonstrated efficacy in reversing antibiotic-induced dysbiosis by reintroducing keystone taxa and metabolites. Recent research confirms FMT rapidly reverses antibiotic-induced dysbiosis in fish by restoring aromatic amino acid metabolism and glutathione synthesis—critical for mucosal repair—while accelerating recovery 2.5-fold compared to natural restoration (Han Z. et al., 2024). For example, florfenicol-treated koi carp (*Cyprinus carpio*) exhibited reduced beneficial genera (e.g., *Lactobacillus*, *Bifidobacterium*) and mucosal damage, but FMT rapidly restored these populations and normalized critical metabolites like aromatic amino acids and glutathione, accelerating recovery compared to natural restoration (Han Z. et al., 2024). FMT also restores key metabolites, including short-chain fatty acids and lipid metabolism-related molecules, supporting a balanced metabolic profile (Xiao et al., 2020; Han Z. et al., 2024). Gut microbiota-derived metabolites like indole 3-propionic acid further demonstrate non-gut protective roles, such as radiation toxicity mitigation (Xiao et al., 2020). Similarly, FMT in large yellow croaker (*Larimichthys crocea*) larvae improved growth performance, digestive enzyme activity, and intestinal morphology by enhancing microbial diversity and introducing functional taxa linked to nutrient metabolism (Zhang et al., 2023). Notably, FMT can transfer phenotype-specific effects: Transplantation of microbiota from antipsychotic-exposed common carp induced behavioral abnormalities in recipient fish, confirming causal microbiota-host nervous system interactions (Chang et al., 2024). This supports evidence that FMT can transfer non-gut traits like behavior and stress tolerance in aquatic animals (Ouyang et al., 2023), and disease susceptibility/resistance between species (Ma et al., 2022). A meta-analysis revealed that higher donor strain engraftment significantly correlates with clinical success (*p* = 0.017) (Ianiro et al., 2022). The study found that species from the phyla *Bacteroidota* (e.g., genera like *Bacteroides* and *Parabacteroides*) and *Actinobacteria* (e.g., families like *Bifidobacteriaceae* and *Coriobacteriaceae*) demonstrated significantly higher engraftment success than most species from the *Firmicutes* phylum. This underscores the critical role of recruiting specific, highly engraftable taxa in achieving ecological resilience and clinical efficacy after FMT. Beyond restoration, early-life microbial interventions can induce durable and even *intergenerational* protection, as demonstrated in the Pacific oyster (*Crassostrea gigas*), where microbial exposure protected against Pacific Oyster Mortality Syndrome (POMS) across generations via epigenetic reprogramming and sustained immune gene expression (Fallet et al., 2022). Early-life FMT modulates immune programming and may enhance disease resistance, though epigenetic mechanisms and cross-generational evidence specifically from FMT remain emerging areas (Cao et al., 2023; Dong et al., 2024).

### 4.2 Synthetic microbial communities (SynComs)

SynComs transcend restoration by enabling targeted manipulation of host functions through ecological and functional strain selection. The advanced design incorporates AI-predicted strain interactions and CRISPR-enhanced traits (e.g., bile tolerance), validated within *in vitro* gut-on-chip systems—microfluidic devices that culture intestinal epithelial cells under controlled flow with co-cultured microbes to emulate gut physiology—before deployment (Guo et al., 2023). Gnotobiotic models have been pivotal in elucidating strain-specific roles, such as *Cetobacterium* dominance in probiotic-fed grey mullet correlating with upregulated immune genes (*IL-1β*, *TNF-α*) and improved survival against *Nocardia seriolae* (Chan et al., 2024). Rational design is exemplified by a shrimp SynCom (*Paracoccus*, *Ruegeria*, *Microbacterium*, *Demequina*, *Tenacibaculum*) that suppressed *V. parahaemolyticus*, improved growth, and restored immune parameters through competitive exclusion and metabolic synergy (Guo et al., 2023). This SynCom reduced pathogenic *Vibrio* by 89% and enhanced thermal resilience via *Tenacibaculum*-mediated metabolic adjustments (Guo et al., 2023). SynComs can also improve environmental resilience (e.g., thermal/salinity tolerance) via optimized resource utilization, member replacement, and microbial communication (Jiang et al., 2024; Dubey et al., 2025). Case studies highlight their precision: a *Bacillus* spp. and *Lactobacillus plantarum* mix reduced *Streptococcus agalactiae* mortality by 40% in Nile tilapia via gut barrier enhancement (Huang X. et al., 2024), while *Streptomyces* sp. D6 suppressed *Aeromonas veronii* in crucian carp using bacteriocin-like compounds (Zhao et al., 2025). Encapsulation technologies further optimize SynCom delivery by enhancing microbial persistence in hydrogels (Fu S. et al., 2025).

Both strategies require rigorous biosafety frameworks due to inherent risks. FMT may facilitate horizontal gene transfer (HGT) of antibiotic resistance or virulence factors, particularly if donors harbor dysbiotic or pro-inflammatory communities (e.g., carrageenan-induced microbiota exacerbating colitis) (Wu et al., 2021, 2022). Engraftment failure is common when recipient conditions (e.g., diet, water chemistry) mismatch donor niches, as shown in interspecific transplants where dietary alignment was critical for stability (Li W. J. et al., 2022; Ruiz et al., 2024). Multi-omics-guided keystone taxon identification (e.g., *Akkermansia* for mucus integrity) minimizes ecological disruption during transplantation (Huang et al., 2025). For SynComs, an incomplete understanding of strain interactions risks community collapse or dominance of opportunistic taxa. Furthermore, environmental persistence of introduced strains could disrupt native microbiomes or nutrient cycles (Zhao Z. et al., 2023). Standardized donor screening, multi-omics-guided keystone taxon identification, and ecological risk assessments are thus essential for responsible application.

## 5 Emerging tools and technologies

CRISPR and AI further refine precision by enabling targeted trait augmentation in probiotics and predictive optimization of SynComs, closing the loop between multi-omics discovery and intervention design. Together with gut-on-chip platforms and *in vitro* models, these tools form a unified technological framework enabling a cohesive closed-loop engineering cycle where experimental validation informs AI-driven SynCom design and CRISPR-based refinement. This synergy transforms fragmented approaches into a cohesive pipeline for precision interventions. The frontier of precision microbiome engineering is defined by an integrated technological framework where in vitro models, computational tools, genome editing, and synthetic ecology converge to enable iterative design-test-deploy cycles.

### 5.1 Gut-on-chip platforms

Gut-on-chip platforms—advanced microfluidic devices that emulate the structural complexity, cellular organization, and dynamic physiology of the intestinal tract—are revolutionizing the study of host–microbe interactions by simulating the intestinal microenvironment of aquatic species. These systems recreate critical features including mucus-secreting epithelia, vascular-like perfusion, mechanical peristalsis, and oxygen gradients, enabling physiologically relevant modeling of gut barrier integrity, immune responses, and probiotic colonization dynamics. By integrating living cells from target species (e.g., fish intestinal epithelium), these chips allow precise testing of probiotic efficacy, host-pathogen interactions, and metabolite exchange under controlled yet dynamic conditions. This technology bridges the gap between traditional in vitro models (oversimplified) and *in vivo* trials (ethically and logistically challenging), offering high-throughput screening of microbiome interventions. For example, a recent dual-sample microfluidic LAMP ‘gut-on-chip’ detects ten aquatic pathogens—including *Vibrio parahaemolyticus*—within 30 min (93% clinical sensitivity), providing a rapid pathogen-challenge module that can be coupled to probiotic screening workflows (Zhou et al., 2021). Integration with lateral flow assays (μLAMP-LFA) further enhances field applicability by enabling multiplexed diagnostics while preventing aerosol contamination (Zhu et al., 2025). Gnotobiotic models, such as germ-free zebrafish (Solis et al., 2020; Jia et al., 2024), have laid the groundwork for these platforms by demonstrating how specific bacterial communities influence host immunity and pathogen resistance. For instance, germ-free zebrafish colonized with synthetic microbial communities (SynComs) revealed that commensal bacteria like *Cetobacterium somerae* enhance glucose homeostasis via acetate production (Wang et al., 2021), insights critical for validating Gut-on-Chip responses. Recent studies using germ-free Atlantic salmon (Gómez de la Torre Canny, 2023) further highlight how mucosal barrier function and adipose tissue dynamics depend on microbiota composition, parameters that can be monitored *in vitro* using chip technology. By integrating multi-omics data from gnotobiotic models, Gut-on-Chip systems allow high-throughput screening of probiotics, reducing reliance on live animal tria0-9ls while accelerating the development of targeted microbial therapies. These platforms thus serve as physiologically relevant validation hubs within the engineering cycle.

### 5.2 Artificial intelligence and machine learning

Artificial intelligence (AI) and machine learning (ML) are transforming microbiome engineering by predicting host–microbe interactions and optimizing probiotic formulations through the integration of complex multi-omics datasets, environmental metadata, and phenotypic traits. For instance, support vector machines (SVM) and random ferns (RFerns) achieved 100% accuracy in detecting water quality parameters linked to aquaculture disease outbreaks (Çakir et al., 2023), while conditional forest and random forest algorithms accurately predicted *Salmonella* contamination in agricultural waters using microbiome signatures (Chung et al., 2023). Neural networks discriminated probiotics from non-probiotics with >90% accuracy by analyzing tRNA information content (Bergamini et al., 2022), and decision tree models predicted the *in vivo* immunomodulatory activity of lactic acid bacteria in snails with 88% accuracy by prioritizing phenotypic traits like hydrophobicity and autoaggregation (Charizani et al., 2024). Meta-analyses of zebrafish gut microbiota have identified stage-specific microbial taxa, data that can train ML algorithms to design age-specific probiotics (Garibay-Valdez et al., 2024). AI-driven tools like VirOncoTarget refine pathogen risk assessment by screening viral oncoproteins via adversarial networks (98% accuracy) (Beltrán et al., 2024), while ensemble spatial models predict methane emissions by correlating microbial taxa with environmental stressors (Chen C. C. et al., 2025). Bayesian networks (e.g., SAMBA tool) model how farming conditions alter gut microbiome diversity and predict responses to environmental shifts (Soriano et al., 2023). In shrimp aquaculture, SynComs enriched with *Paracoccus* and *Ruegeria* to suppress *Vibrio parahaemolyticus*, a process optimizable via ML-predicted synergistic combinations. Similarly, Stagaman et al. (2024) linked benzo[a]pyrene toxicity to microbiome diversity in zebrafish, demonstrating ML’s capacity to correlate pollutants with dysbiosis. ML also models climate impacts, such as warming-driven enterotype migration in *Litopenaeus vannamei* to forecast disease risk (Zeng et al., 2025). AI-driven resources like Microbiome Atlas (Lin et al., 2021) catalog bacterial growth patterns for colonization modeling, while foundation models with transfer learning adapt predictions to specific aquaculture contexts despite data sparsity (Han et al., 2025). These approaches are critical for aquaculture, where dynamic conditions demand adaptive strategies. Collectively, AI/ML provides the computational engine for *in silico* design and optimization of microbial consortia before empirical testing, enabling precision modulation of microbiomes for disease prevention, growth enhancement, and environmental resilience.

### 5.3 CRISPR-based genome editing

CRISPR-based genome editing is emerging as a powerful tool to engineer probiotics with enhanced pathogen-inhibiting capabilities. Recent research demonstrates how engineered riboregulators and CRISPR-based devices can enhance auxotrophic biocontainment in genetically modified probiotics. For instance, Cas9-assisted containment systems in *Bacteroides thetaiotaomicron* blocked transgene dissemination and prevented escape via thymidine auxotrophy, establishing a model for biosafety in aquaculture-engineered strains (Hayashi et al., 2024). Applications include CRISPR-Cas9 knockout of the *hfq* gene in *Vibrio alginolyticus*, reducing motility and mortality in scallops by disrupting biofilm formation (Ma et al., 2023), and Cas13a-mediated targeting of white spot syndrome virus (WSSV) in shrimp, extending survival by 33% post-infection (Pudgerd et al., 2024). Regulatory frameworks for CRISPR applications in aquaculture, particularly in the EU and Asia, are evolving to address biosafety and horizontal gene transfer risks, as highlighted in recent policy reviews (Gutási et al., 2023). Furthermore, phage-plasmid systems encoding toxin-antitoxin modules and CRISPR-Cas systems have been shown to limit horizontal gene transfer, enhancing host microbial immunity (Sayid et al., 2024). Direct host genome editing has also shown promise: CRISPR-modified tilapia exhibited enhanced resistance to *Streptococcus agalactiae* by targeting immune pathways like TLR2 (Yang et al., 2022), highlighting dual strategies for microbiome and host engineering. For instance, *Aeromonas veronii* secretes GlcNAc-binding protein (GbpA), which stimulates intestinal epithelial proliferation in zebrafish (Banse et al., 2023). CRISPR could augment GbpA expression or modify adhesion factors to improve probiotic persistence. Similarly, *Cetobacterium somerae*, which activates TLR2-mediated antiviral immunity (Liang et al., 2024), might be engineered to overexpress immunostimulatory exopolysaccharides. While CRISPR applications in aquaculture remain nascent, studies on *Vibrio* sp. and *Aeromonas* sp. (Xin et al., 2020) demonstrate how innate immune pathways can be targeted via genetically modified microbes. Challenges include ensuring horizontal gene transfer prevention and addressing regulatory concerns, but CRISPR’s precision offers unparalleled potential for tailored microbial solutions. This technology enables precise trait augmentation in probiotic chassis, feeding directly into AI-designed SynCom blueprints.

### 5.4 Climate-resilient strategies

Rising water temperatures threaten aquaculture productivity, necessitating probiotics that thrive under thermal stress. A case study by Guo et al. (2023) illustrates how SynComs designed for shrimp resilience can be adapted using ML. By analyzing microbiota from biofloc systems, their team identified heat-tolerant taxa like *Tenacibaculum*, which were incorporated into SynComs to enhance *Vibrio* resistance. Biopolymer encapsulation (e.g., alginate-microencapsulated *Bacillus licheniformis*) ensures >90% gastric viability and sustained function across temperature fluctuations (Cota-Gastélum et al., 2025), while directed evolution yields strains like *Bacillus subtilis* TLDK301120C24, validated in gnotobiotic zebrafish to displace pathogens via competitive biofilm exclusion (Nayak et al., 2023). Such climate-resilient strategies align with SDG 13 (Climate Action), as highlighted in Table 3, which maps microbiome engineering approaches to their contributions toward mitigating environmental stressors. ML algorithms can further optimize these communities by predicting strain interactions under simulated warming scenarios. For example, *Cetobacterium*, a dominant genus in zebrafish guts (Garibay-Valdez et al., 2024), shows temperature-dependent acetate production (Wang et al., 2021), a trait ML could exploit to design climate-resilient consortia. For cold-water species like olive flounder (*Paralichthys olivaceus*), AI-guided SynComs enriched with *Pseudomonas* and *Comamonas* restore PUFA synthesis at suboptimal temperatures, countering metabolic dysregulation (Chen C. C. et al., 2025). Integrating environmental metagenomics with host transcriptomic data (Andersen-Civil et al., 2023) will refine these models, enabling probiotics that buffer against both pathogen outbreaks and climate variability. Deploying these advances on working farms, however, brings regulatory, ecological, and socioeconomic hurdles into sharp relief.

**Table 3:** Microbiome engineering strategies aligned with un sdgs to enhance disease resistance in aquaculture.

SDG	Relevance to disease resistance	Strategy	Example application	Key outcomes	References
SDG 2	Improve nutrient absorption to reduce stress-induced disease susceptibility.	Probiotic *Bacillus subtilis* LCBS1 cell wall derivatives.	Bullfrog (*Aquarana catesbeianus*) fed soybean meal.	Reduced intestinal inflammation, enhanced gut barrier function.	Tao et al. (2024)
SDG 3	Suppress antibiotic-resistant pathogens via immunomodulation.	Heat-killed *Lactiplantibacillus plantarum* (HK L-137).	Atlantic salmon (*Salmo salar*) challenged with *Vibrio*.	Increased plasma IgM, reduced oxidative stress, and balanced gut microbiota.	Rocha et al. (2023)
SDG 6	Reduce pathogen spread through wastewater bioremediation.	*Streptomyces rochei* ANH for heavy metal removal.	Tannery effluent treatment for aquaculture reuse.	86% Cr^6+^ removal, improved water safety.	Hamdan et al. (2021)
SDG 13	Mitigate climate-driven pathogen proliferation.	Microalgae blend (*Tisochrysis lutea*, *Nannochloropsis gaditana*).	Gilthead seabream (*Sparus aurata*) fed algal diets.	Reduced methane emissions, and enhanced lipid metabolism.	García-Márquez et al. (2023)
SDG 14	Combat marine pathogen outbreaks (e.g., *Vibrio*).	*Bacillus* sp. KRF-7 isolated from rockfish intestines.	Rockfish (*Sebastes schlegelii*) challenged with *Vibrio*.	Increased survival, reduced pathogen load, and *Bacillota* dominance.	Jang et al. (2021)
SDG 17	Accelerate vaccine development through shared data.	Transcriptomic analysis of *Nocardia seriolae*-infected hybrid snakehead.	Identification of immune biomarkers for vaccine design.	Improved pathogen surveillance and vaccine targeting.	Zhang et al. (2022)
SDG 2	Enhance aquaculture production for food security.	Probiotics and biofloc technology	Nile tilapia (*Oreochromis niloticus*) in biofloc system.	Improved growth, microbial community composition, and water quality.	Oliveira et al. (2025)
SDG 3	Reduce gut inflammation and improve immunity.	Enzymatically hydrolyzed compound soy protein	Juvenile American eel (*Anguilla rostrata*) fed diets containing graded levels (0–32%) of EHCS, with optimal results at ~8%, for 10 weeks	Enhanced growth, improved intestinal health, and probiotic balance.	Xu et al. (2024)
SDG 14	Improve intestinal health and immunity.	Postbiotics from (*Bacillus suBS1*)	Bullfrog (*Aquarana catesbeianus*) fed soybean meal.	Reduced intestinal inflammation, enhanced immune response.	Tao et al. (2024)
SDG 14	Improve growth and immunity through dietary supplementation acid-producing additives	Sea cucumber (*Apostichopus japonicus*) fed butyrate.	Sea cucumbers were fed a diet supplemented with butyrate.	Enhanced growth, increased immune response, and better gut microbiota.	Liu L. et al. (2024)

### 5.5 Synergistic closed-loop engineering cycle

These technologies form a closed-loop engineering cycle: AI/ML leverages multi-omics data to design optimized SynComs; CRISPR introduces precision traits into probiotic chassis; Gut-on-Chip platforms validate host–microbe interactions under simulated environmental conditions; and encapsulation ensures field resilience. For instance, AI-designed *Vibrio*-targeting consortia can be genome-edited for enhanced bile tolerance, functionally validated in microfluidic intestines, and deployed via temperature-stable encapsulates (Vijayaram et al., 2024; Ding et al., 2025). This iterative workflow bridges computational prediction with empirical delivery, establishing a scalable framework for precision microbiome engineering in aquaculture.

## 6 Current status of practical applications in aquaculture

Translating microbiome engineering innovations from laboratory concepts to commercial aquaculture practice reveals a continuum of adoption. Probiotics and synbiotics represent the most mature and widely implemented tools, while postbiotics, fecal microbiota transplantation (FMT), synthetic microbial communities (SynComs), artificial intelligence/machine learning (AI/ML) decision-support, and CRISPR-enabled interventions face distinct stages of field validation and regulatory hurdles. Success hinges on aligning strain functionality with host ecology, environmental parameters, and socioeconomic contexts (Guo et al., 2023; El-Son et al., 2025).

### 6.1 Commercial availability and on-farm performance

Probiotic formulations—particularly resilient *Bacillus* spp.—dominate commercial adoption in major aquaculture regions like Asia-Pacific, where they competitively exclude pathogens (*Vibrio*, *Aeromonas*, *Edwardsiella*) via quorum-quenching mechanisms and enhance digestive physiology (Muras et al., 2021; Santos et al., 2021). EU-approved probiotics (e.g., *Pediococcus acidilactici*) demonstrate immune modulation in salmonids, reducing mortality from pathogens like *Yersinia ruckeri* by 25–50% (Villumsen et al., 2020; Yousuf et al., 2023). Synbiotics show accelerated growth, with shrimp-specific formulations improving yields by >600 kg/pond through ammonia reduction and microbiome stabilization (Shinde et al., 2023; Wang Q. et al., 2024). Postbiotics gain traction for storage stability and reduced regulatory burdens; heat-inactivated *Lactiplantibacillus plantarum* and engineered *Lactococcus lactis* (expressing host cytokines) enhance immune responses and disease resistance in tilapia and salmon without live-cell risks (Muñoz et al., 2021; Ferro et al., 2024). Plant-derived supplements (e.g., fermented herbal blends, resveratrol) and encapsulated bioactives (e.g., nano-astaxanthin) further augment growth and stress tolerance, though adoption varies regionally (Fujaya et al., 2023; Elbahnaswy and Elshopakey, 2024; Kari et al., 2024; Lee et al., 2024).

### 6.2 Field trials and species-specific efficacy

Field validations confirm significant productivity gains: *Bacillus velezensis* supplementation in shrimp ponds increased survival by 23% and upregulated hepatopancreatic immune genes (Abdelsamad et al., 2024), while synbiotic strategies in giant freshwater prawns induced complete resistance to *Aeromonas veronii* (Chin et al., 2025). Postbiotics accelerated microbiome recovery post-antibiotics in shrimp, reducing pathogenic *Vibrio* by 40% (Luo et al., 2024b). Species-tailored blends (e.g., *Pediococcus*/*Lactococcus*/*Weissella* consortia in trout) enhanced intestinal morphology and cytokine expression (Mahmoodian et al., 2025). Performance gains are most consistent when formulations align with host ecology—e.g., temperature-adapted probiotics in olive flounder improved winter survival (Lee et al., 2024), and microalgae-phytase synergies boosted seabass growth without disrupting core microbiota (Peralta-Sánchez et al., 2024).

### 6.3 Emerging tools: SynComs and FMT

Rational SynComs designed from native taxa show promise in pathogen suppression (e.g., *V. parahaemolyticus* in shrimp) and stress resilience (e.g., salinity/thermal tolerance) but remain at the pilot stage due to challenges in donor-recipient compatibility and ecological instability (Wang Z. et al., 2023). FMT effectively restores antibiotic-disrupted microbiomes in controlled settings yet faces barriers in horizontal gene transfer (HGT) risk (e.g., antimicrobial resistance (AMR) propagation) and farm-scale workflow standardization (Behling et al., 2024; Tian et al., 2024). Encapsulation (e.g., pH-responsive microcapsules) is critical for SynCom viability and intestinal delivery, though ecological matching and gnotobiotic validation are prerequisites for field success (Dremova et al., 2023; Jiang et al., 2024).

### 6.4 AI/ML and CRISPR: readiness and constraints

AI/ML tools enable risk prediction and *in silico* consortium design but require integration into telemetry-linked farm trials to address domain shifts between lab and pond conditions (Bergamini et al., 2022; Çakir et al., 2023). CRISPR applications (e.g., containment-enhanced probiotics, host-immunity tuning) are constrained by regulatory ambiguity—particularly GMO classification in the EU—and unresolved technical hurdles like off-target effects and biocontainment validation (Okoli et al., 2022; Gutási et al., 2023; Sheng et al., 2025). Field-validated environmental DNA (eDNA) surveillance (Technology Readiness Level [TRL] 6) demonstrates utility in species detection but lacks standardized ecosystem integration (Kim et al., 2024).

## 7 Challenges and future directions

Technical barriers center on strain-specific complexities, where probiotic efficacy hinges critically on precise selection and dosing. For instance, while low-dose *Bacillus subtilis* enhances intestinal health in Chinese perch (*Siniperca chuatsi*), higher concentrations impair growth, and antimicrobial peptides exhibit variable efficacy across species due to divergent host physiologies and environmental conditions (Ji et al., 2023; Wang J. et al., 2023). Host-microbiome compatibility further complicates design, as evidenced by germ-free rainbow trout colonized with protective *Flavobacterium* spp. resisting *Flavobacterium columnare* infection, whereas mismatched consortia increase mortality (Perez-Pascual et al., 2021).

Ecological risks arise from introducing non-native strains, particularly HGT of antibiotic resistance genes (ARGs). Conjugative transfer rates in aquaculture settings range from 10^−5^ to 1%, amplified by environmental stressors like triclosan, which increases ARG transfer frequencies by 1.2–1.4-fold in *Edwardsiella piscicida* (Lu et al., 2022). Examples include *aphA* in sphingomonads and *floR* in *Vibrio parahaemolyticus*, both contributing to ARG dissemination (Fu et al., 2022; Qian et al., 2024). Engineered probiotics (e.g., CRISPR-modified *Bacillus subtilis*) may persist in sediments, disrupting nitrogen cycles or outcompeting keystone species (Yang et al., 2024; Lee et al., 2025). Mitigation requires CRISPR-based biocontainment (e.g., thymidine auxotrophy “kill switches”) (Lee et al., 2025), supplemented by a tiered ecological monitoring framework: (1) quantitative eDNA metabarcoding to track ARG dissemination (e.g., sul1, aadA1) at sensitivities of 10^6^ copies/L across water/sediment matrices (Vandeputte et al., 2017; Zhang Y. et al., 2025); (2) stressor-responsive mesocosm trials evaluating HGT frequencies under heavy metal/pH fluctuations (Yue et al., 2023; Hazra et al., 2024); and (3) mandatory surveillance of mobile genetic elements (intI1) in effluents integrated with biocontrol alternatives like Traditional Chinese Medicine (40–60% ARG reduction) (Wang et al., 2018; Li et al., 2024).

Regulatory fragmentation impedes global deployment. The EU’s stringent pre-market assessments (Directive 2001/18/EC) contrast with ASEAN’s voluntary standards and China’s rapid but less-regulated adoption, resulting in trade disruptions—35% of genetically modified feed imports from China were rejected by the EU’s Rapid Alert System for unresolved ecological risks (Eissa et al., 2024). Harmonizing frameworks through FAO/WHO guidelines for strain-specific safety evaluations and incentivizing alternatives (e.g., chitooligosaccharides) (Mohan et al., 2023) are essential to bridge these disparities. These efforts must prioritize standardization of the ecological monitoring framework (eDNA, mesocosms, MGE surveillance) under One Health-aligned protocols to ensure global consistency in detecting unintended impacts (Arnold et al., 2024). Moreover, CRISPR/Cas-based genome editing introduces new uncertainties around off-target effects and trait predictability, which remain insufficiently addressed in current EU and international policies (Okoli et al., 2022).

Socioeconomic barriers include high production costs of synthetic probiotics and limited adoption in resource-poor regions. In the Indian Sundarbans, trained farmers using probiotics achieved a 66% higher benefit–cost ratio than traditional practices, yet adoption remains constrained by inadequate institutional support and financial access (Ghosh et al., 2022). Many operators also continue to prefer traditional antibiotics despite their ecological risks, indicating a need for improved training and awareness programs (He et al., 2022). Bridging this gap demands democratizing cost-effective solutions—such as integrating *Rhodobacter sphaeroides* with biofloc systems to recycle nitrogen waste (Cao et al., 2024)—and fostering ASEAN-China partnerships to align biocontainment standards (Noman et al., 2024).

Addressing these challenges necessitates integrated strategies grounded in ecological theory, including multi-omics-guided formulations (e.g., tannic acid-modulated PPAR pathways in turtles) (Ji et al., 2025), K-selection-based community management (Vadstein et al., 2018), circular aquaculture systems valorizing waste, and AI-optimized climate-resilient SynComs. By aligning with UN Sustainable Development Goals—particularly Zero Hunger (SDG 2), Climate Action (SDG 13), and Life Below Water (SDG 14)—microbiome engineering can mitigate antimicrobial resistance while securing ecologically balanced aquaculture.

## 8 Conclusion

Microbiome engineering represents a transformative approach anchored in precision tools. Multi-omics profiling identifies host-specific biomarkers, informing the design of probiotics, synbiotics, FMT, and SynComs. CRISPR-edited probiotics and AI-driven SynCom optimization then enable targeted, climate-resilient interventions by predictively manipulating host-microbe-environment interactions. Together, these strategies enhance disease resistance while reducing antibiotic reliance. Advanced tools such as CRISPR-engineered probiotics and AI-driven SynCom design further refine these interventions, enabling precise modulation of host–microbe interactions under climate stressors. For instance, *Bacillus subtilis* strains engineered to express pathogen-specific antigens and heat-tolerant SynComs incorporating *Tenacibaculum* exemplify the potential of these innovations to bolster resilience in dynamic environments. However, scaling these solutions requires overcoming critical challenges, including strain-host compatibility, horizontal gene transfer (HGT) risks, regulatory disparities, and socioeconomic barriers. Collaborative efforts to harmonize global policies—such as adopting FAO/WHO safety frameworks—and democratize access to cost-effective alternatives (e.g., chitooligosaccharides and biofloc systems) are essential. Future advancements require tailored farm-specific probiotics designed via multi-omics profiling, circular aquaculture systems integrating waste valorization, and climate-resilient SynComs optimized via machine learning. Global policies must align with the United Nations Sustainable Development Goals (SDGs)—Zero Hunger (SDG 2), Climate Action (SDG 13), and Life Below Water (SDG 14)—to ensure equitable adoption and ecological safety. Aligning with these SDGs can mitigate antimicrobial resistance (AMR), restore aquatic biodiversity, and secure food production for a growing population. To realize this vision, interdisciplinary collaboration among researchers, policymakers, and industry stakeholders is urgent. Only through coordinated innovation and equitable implementation can aquaculture transition from a sector burdened by ecological trade-offs to a model of sustainable food systems.
